# ^18^F-Fluorocholine PET/CT in the assessment of primary hyperparathyroidism compared with ^99m^Tc-MIBI or ^99m^Tc-tetrofosmin SPECT/CT: a prospective dual-centre study in 100 patients

**DOI:** 10.1007/s00259-018-3980-9

**Published:** 2018-03-08

**Authors:** Mohsen Beheshti, Lukas Hehenwarter, Zeinab Paymani, Gundula Rendl, Larisa Imamovic, Rupert Rettenbacher, Oleksiy Tsybrovskyy, Werner Langsteger, Christian Pirich

**Affiliations:** 1 Department of Nuclear Medicine & Endocrinology, PET-CT Center Linz, Ordensklinikum, St. Vincent’s Hospital, Linz, Austria; 2 Department of Nuclear Medicine & Endocrinology, Paracelsus Medical University Salzburg, University Hospital, Müllner Hauptstraße 48, A-5020 Salzburg, Austria; 3 Research Center for Nuclear Medicine, Shariati Hospital, Tehran University of Medical Sciences, Tehran, Iran; 4 Department of Surgery, Paracelsus Medical University, Salzburg, Austria; 5 Department of Clinical Pathology, Ordensklinikum, St. Vincent’s Hospital, Linz, Austria

**Keywords:** ^18^F-Fluorocholine PET/CT, Primary hyperparathyroidism, ^99m^Tc-MIBI, ^99m^Tc-Tetrofosmin, SPECT/CT

## Abstract

**Purpose:**

In this prospective study we compared the accuracy of ^18^F-fluorocholine PET/CT with that of ^99m^Tc-MIBI or^99m^Tc-tetrofosmin SPECT/CT in the preoperative detection of parathyroid adenoma in patients with primary hyperparathyroidism. We also assessed the value of semiquantitative parameters in differentiating between parathyroid hyperplasia and adenoma.

**Methods:**

Both ^18^F-fluorocholine PET/CT and ^99m^Tc-MIBI/tetrofosmin SPECT/CT were performed in 100 consecutive patients with biochemical evidence of primary hyperparathyroidism. At least one abnormal focus on either ^18^F-fluorocholine or ^99m^Tc-MIBI/tetrofosmin corresponding to a parathyroid gland or ectopic parathyroid tissue was considered as a positive finding. In 76 patients with positive findings on at least one imaging modality, surgical exploration was performed within 6 months, and the results were related to histopathological findings and clinical and laboratory findings at 3–6 months as the standard of truth. In 24 patients, no surgery was performed: in 18 patients with positive imaging findings surgery was refused or considered risky, and in 6 patients imaging was negative. Therefore, data from 82 patients (76 undergoing surgery, 6 without surgery) in whom the standard of truth criteria were met, were used in the final analysis.

**Results:**

All patients showed biochemical evidence of primary hyperparathyroidism with a mean serum calcium level of 2.78 ± 0.34 mmol/l and parathormone (PTH) level of 196.5 ± 236.4 pg/ml. The study results in 76 patients with verified histopathology and 3 patients with negative imaging findings were analysed. Three of six patients with negative imaging showed normalized serum PTH and calcium levels on laboratory follow-up at 3 and 6 months, and the results were considered true negative. In a patient-based analysis, the detection rate with ^18^F-fluorocholine PET/CT was 93% (76/82), but was only 61% (50/82) with ^99m^Tc-MIBI/tetrofosmin SPECT/CT. In a lesion-based analysis, the sensitivity, specificity, positive predictive value, negative predictive value and overall accuracy of ^18^F-fluorocholine PET/CT in the detection of parathyroid adenoma were 93.7%, 96.0%, 90.2%, 97.4% and 95.3%, respectively, and of ^99m^Tc-MIBI/tetrofosmin SPECT/CT were 60.8%, 98.5%, 94.1%, 86.3% and 87.7%, respectively. Although ^18^F-fluorocholine PET-positive adenomatous lesions showed higher SUVmax values than the hyperplastic glands (6.80 ± 3.78 vs. 4.53 ± 0.40) in the semiquantitative analysis, the difference was not significant (*p* = 0.236). The mean size (measured as the length of the greatest dimension) and weight of adenomas were 15.9 ± 7.6 mm (median 15 mm, range 1–40 mm) and 1.71 ± 1.86 g (median 1 g, range: 0.25–9 g), respectively. Among the analysed parameters including serum calcium and PTH and the size and weight of parathyroid adenomas, size was significantly different between patients with negative ^99m^Tc-MIBI/tetrofosmin SPECT/CT and those with positive ^99m^Tc-MIBI/tetrofosmin SPECT/CT (mean size 13.4 ± 7.6 mm vs. 16.9 ± 7.4 mm, respectively; *p* = 0.042).

**Conclusion:**

In this prospective study, ^18^F-fluorocholine PET/CT showed promise as a functional imaging modality, being clearly superior to ^99m^Tc-MIBI/tetrofosmin SPECT/CT, especially in the detection and localization of small parathyroid adenomas in patients with primary hyperparathyroidism. SUVmax was higher in parathyroid adenomas than in hyperplasia. However, further evaluation of this modality is needed.

## Introduction

Primary hyperparathyroidism (PHPT) is the most prevalent cause of hypercalcaemia in outpatients [1]. PHPT is induced most frequently by a solitary parathyroid adenoma (80–85%) followed by four-gland hyperplasia (10–15%) and double adenoma (4%) [2]. The current way of treating symptomatic or young patients (<50 years of age) is surgical resection of the underlying adenoma [3]. Image-guided minimally invasive parathyroidectomy is preferred over classic bilateral neck exploration since it shows similar efficacy and is associated with less discomfort due to a shorter excision, shorter operation time, fewer complications, faster recovery and overall less expense for the patient [3, 4]. The minimally invasive surgical approach is planned following preoperative detection and localization of all causative adenomas using an accurate imaging modality [5]. Several imaging procedures have been proposed for the detection and localization of parathyroid adenomas. These include anatomical techniques such as ultrasonography, computed tomography (CT) and magnetic resonance imaging (MRI), and hybrid functional imaging techniques such as ^99m^Tc-MIBI single photon emission computed tomography (SPECT) in combination with CT (SPECT/CT) [3, 4].

^99m^Tc-labelled methoxy-isobutyl-isonitrile (MIBI) and tetrofosmin are both lipophilic cations that concentrate in the mitochondria corresponding to the transmembrane electrical potential and have shown close biological behaviour and detection values in clinical trials [6–9]. ^99m^Tc-MIBI SPECT has long been used and is considered the imaging modality of choice for the detection and localization of parathyroid adenomas [4, 10]. Studies have shown variable accuracy for ^99m^Tc-MIBI SPECT with equivocal imaging results in nearly one third of patients [11]. Recent meta-analyses have shown higher detection rates of 84–88% (in both patient-based and lesion-based analyses) for hybrid ^99m^Tc-MIBI SPECT/CT imaging [6, 12, 13]. Recently, the role of positron emission tomography/computed tomography (PET/CT) imaging using specific PET tracers has been highlighted. This approach provides complementary anatomical and functional information and exhibits higher spatial and temporal resolution [5].

Several PET radiotracers have been introduced in recent years for the assessment of PHPT. The most commonly used PET tracer is ^11^C-methionine. However, the value of ^18^F-fluorocholine, a marker of cellular proliferation, in the detection and localization of parathyroid adenoma has recently been shown [5]. ^18^F-Fluorocholine has a longer half-life and provides more flexibility for patient management in PET/CT centres. Previous studies have shown promising results with ^18^F-fluorocholine PET/CT in the assessment of PHPT. This prospective study was designed to compare the value of ^18^F-fluorocholine PET/CT with that of ^99m^Tc-MIBI or ^99m^Tc-tetrofosmin SPECT/CT in detecting parathyroid adenomas in a large patient population and to correlate the results of the imaging modalities with histopathological findings and laboratory parameters on follow-up examinations. This work also assessed the value of semiquantitative parameters in differentiating between parathyroid hyperplasia and adenoma.

## Materials and methods

### Patients

In this prospective dual-centre study, 100 patients (21 men, 79 women; mean age 57.4 ± 12.5 years) with clinical evidence of PHPT, i.e. those with elevated serum levels of parathormone (PTH) and/or calcium, were enrolled from October 2015 to October 2017. The inclusion criteria were clinical evidence of PHPT and ability to tolerate both ^99m^Tc-MIBI or ^99m^Tc-tetrofosmin SPECT/CT and ^18^F-fluorocholine PET/CT. The exclusion criteria were low serum vitamin D levels, other metabolic diseases, known cancer, clinical or laboratory findings suggesting secondary or tertiary hyperparathyroidism, or active inflammatory or granulomatous diseases. The patients underwent imaging with both modalities including ^18^F-fluorocholine PET/CT and ^99m^Tc-MIBI or ^99m^Tc-tetrofosmin SPECT/CT for preoperative assessment and localization of parathyroid adenomas regardless of the ^99m^Tc-MIBI SPECT/CT results.

The study was approved by the institutional Ethics Committee (415-E/1858/2-2015) and was performed according to the principles of the 1964 Declaration of Helsinki and its later amendments or comparable standards. Written informed consent was obtained from all patients.

### Imaging

#### ^18^F-Fluorocholine PET/CT

The study was performed using two different PET/CT scanners. A dedicated PET/CT scanner (Discovery 710; GE Healthcare) consisting of an extended field-of-view full-ring high-resolution LSO PET component and a 128-slice spiral CT component was used in 49 patients. Imaging was performed 60 min after intravenous injection of 3.2 MBq/kg body weight ^18^F-fluorocholine. PET/CT acquisitions were obtained from the base of the skull to the proximal thighs with 2.5 min per bed position using time-of-flight (TOF) mode and a nondiagnostic CT scan (in most patients) for attenuation correction and localization. If tracer uptake in the cervical or thoracic regions was equivocal or unclear, an additional delayed acquisition (100–120 min after injection) was performed. All images were reconstructed identically using the ordered-subsets expectation maximization algorithm (four iterations, 18 subsets) followed by a postreconstruction smoothing gaussian filter (4.0 mm full-width at half-maximum). The slice thickness was 3.75 mm for PET images.

A Philips Ingenuity TF (Philips Healthcare, PC Best, The Netherlands) was used in 33 patients. Imaging was performed 60 min after intravenous injection of 3.2 MBq/kg body-weight ^18^F-fluorocholine. PET/CT acquisitions were obtained from the base of the skull to the diaphragm to include possible ectopic adenomas. Delayed imaging (100–120 min after injection) was obtained if indicated. Images were reconstructed using a three-dimensional ordered-subsets iterative TOF (BLOB-OS-TF) algorithm after correction for scatter and attenuation. For the CT scan (50 mA, 120 kV), a collimation 64 × 0.625 mm, a slice thickness 3 mm and reconstruction increment 1.5 mm were used. The time between the two scans was not more than 2 weeks.

Images were read separately by two experienced nuclear medicine specialists, who were aware of the patient’s clinical diagnosis, previous imaging and laboratory data, using advanced PET/CT review software (Advantage Windows, version 4.6, for the GE Medical Systems scanner; or Philips IntelliSpace, version 8.0, for the Philips Medical Systems scanner) which allowed simultaneous scrolling through the corresponding PET, CT and fusion images in the transverse, coronal and sagittal planes. Images were interpreted separately in each study centre. Any focal ^18^F-fluorocholine uptake above the background tissue uptake was interpreted as pathological hyperfunctioning parathyroid tissue. Lesions were localized anatomically to six regions: right upper, right lower, left upper and left lower thyroid, intrathyroidal, and ectopic (e.g. mediastinal). The thyroid gland showed mild to moderate physiological tracer uptake on ^18^F-fluorocholine PET/CT but this did not affect interpretation of abnormal parathyroid lesions especially when assessing transaxial images.

For semiquantitative analysis, the maximum standardized uptake value (SUVmax) was calculated by manually locating a standard (25 × 25 × 25 mm) volume of interest over the detected pathological lesion related to the parathyroid abnormality on ^18^F-fluorocholine PET/CT images.

The maximum length of the metabolic diameter of the pathological parathyroid lesions was measured manually with a commercial tool provided with the review software.

Neck ultrasonography was performed in all patients for morphological correlation and improvement of image interpretation. However, these data and their analysis are not presented in this article because this was considered beyond the scope of this study.

#### ^99m^Tc-MIBI/tetrofosmin SPECT/CT

Imaging was performed using a dedicated SPECT/CT scanner (Symbia T2 or T6; Siemens Healthineers) after intravenous injection of 740 MBq ^99m^Tc-MIBI (in 49 patients) or ^99m^Tc-tetrofosmin (in 33 patients). Planar imaging of the head/neck and thorax was performed in the anterior and posterior views 15 min, 60 min and 120 min after injection. An additional SPECT/CT acquisition was performed in conjunction with planar 60-min scintigraphy. The SPECT/CT acquisition comprised 64 projections of 20 s each. Images were reconstructed with Flash3D using 14 iterations and four subsets. A low-dose CT scan (130 kV, effective 30 mAs; CARE Dose 4D) was performed for anatomical correlation only.

Images were read using advanced software for multimodality reading (*syngo*.via; Siemens Healthineers). Focal increasing or persistent uptake associated with parathyroid abnormality was considered as pathological hyperfunctioning parathyroid tissue. The lesions were localized to the same six regions as the ^18^F-fluorocholine PET/CT scans.

### Surgery and histopathology

A consensus multidisciplinary meeting was held before surgery and all clinical and imaging findings were reviewed for final diagnosis by a nuclear medicine physician, a radiologist, an endocrinologist and an endocrine surgeon. The surgical approach primarily consisted of focused microinvasive parathyroidectomy (MIP) with intraoperative intact parathormone (iPTH) assay in patients with a single adenoma based on the consensus imaging reading. In patients with multiple parathyroidal lesions, a classical unilateral or bilateral neck exploration with intraoperative iPTH testing was performed. Surgery was considered successful if there was a decrease in iPTH serum levels of at least 50% from the baseline value; otherwise, bilateral classic neck exploration was performed.

Patients with negative imaging were further evaluated by 24-h urine calcium measurement to rule out familial hypocalciuric hypercalcaemia. Serum calcium and PTH levels were determined at 3 and 6 months after surgery or imaging in all patients. All removed parathyroid gland(s) were evaluated histopathologically as the gold standard, including size, weight and the dominant cell type. Parathyroid hyperplasia could only be defined when multiple gland resection had been performed [7]. The size and weight of almost all resected parathyroid glands were measured.

### Statistical analysis

SPSS, version 24.0 for Windows (SPSS Inc. and LEAD Technologies) was used for statistical evaluation of the results. Continuous variables are expressed as mean and median values with standard deviations (SD) as well as minimum and maximum. The normality of distributions was evaluated using the Shapiro-Will test. Student’s *t* test for parametric variables was applied as appropriate to determine the significance of differences in laboratory measurements and SUVs. Categorical variables were evaluated using Fisher’s exact test to compare the accuracies between the two different localization techniques with regard to the correct side of the neck (right or left) and the correct quadrant (right upper, right lower, left upper and left lower) or other more specific locations (i.e. intrathyroidal and mediastinal). *P* values <0.05 were considered statistically significant. Imaging was considered successful if the lesion detected could be anatomically verified and removed on surgery, was histologically confirmed as an adenoma or hyperplasia and the patient was biochemically cured. Findings that could not be histologically verified, or findings that did not result in biochemical cure after surgery to the corresponding site, were considered false-positive.

## Results

Of 100 patients who underwent imaging with both modalities, 18 with positive imaging findings refused surgical resection or had an unexpected clinical event that prevented any intervention. Thus, a total of 82 patients (16 men, 66 women; mean age 59.8 ± 14.8 years) with a mean serum calcium level of 2.78 ± 0.34 mmol/dl (normal range 2.10–2.58 mmol/dl; 28 patients within the normal range) and a mean serum PTH level of 196.5 ± 236.4 pg/ml (normal range 11.1–79.5 pg/ml) were included in the final analysis.

In 76 patients (93%) at least one imaging modality was positive; thus surgical resection was performed. Removal of a single parathyroid gland was performed by MIP in 59 of the 76 patients (78%), and 17 of the 76 patients (22%) underwent multiple gland excision using a classical unilateral or bilateral neck exploration technique. In 6 patients (8%) both ^18^F-fluorocholine PET/CT and ^99m^Tc-MIBI/tetrofosmin SPECT/CT were negative. Three of these patients showed a marked reduction in serum calcium and PTH on the follow-up examinations, and were considered true-negative for statistical analysis. However, in three patients the laboratory parameters showed an increasing pattern and the patients were classified as false-negative. Serum calcium and PTH had normalized at 3 and/or 6 months in all patients who underwent parathyroidectomy.

In the patient-based analysis, ^18^F-fluorocholine PET/CT showed a detection rate of 93% (76 of 82) and ^99m^Tc-MIBI/tetrofosmin SPECT/CT a detection rate of only 61% (50 of 82). Serum calcium and PTH levels were not significantly different between the 76 patients with positive scans and the 6 patients with negative findings on both imaging modalities (Table 1).

**Table 1:** Serum calcium and PTH levels in PET/CT-negative and SPECT/CT-negative patients and in PET/CT-positive and/or SPECT/CT-positive patients

Laboratory parameter	PET/CT-negative and SPECT/CT-negative (*n* = 6)	PET/CT-positive or SPECT/CT-positive (*n* = 76)	*p* value
Calcium (mmol/dl), mean ± SD	2.54 ± 0.20	2.77 ± 0.32	0.088
PTH (pg/ml), mean ± SD	105.6 ± 54.6	174.9 ± 165.3	0.312

The mean size (length of the greatest dimension) and weight of the adenomas were 15.9 ± 7.6 mm (median 15 mm, range 1–40 mm) and 1.71 ± 1.86 g (median 1 g, range 0.25–9 g), respectively. The sizes of the parathyroid adenomas were significantly different between patients negative on ^99m^Tc-MIBI/tetrofosmin SPECT/CT and positive on ^99m^Tc-MIBI/tetrofosmin SPECT/CT. However, there was no meaningful difference between other laboratory and histopathological findings (i.e. serum calcium and PTH levels, and weight of adenoma) between these two groups (Tables 2 and 3).

**Table 2:** Laboratory parameters (serum calcium and PTH) in SPECT/CT-negative patients and SPECT/CT-positive patients, and histopathological findings in the adenomas from the two groups

	SPECT/CT-negative	SPECT/CT-positive	*p* value
Laboratory parameters			
Calcium (mmol/dl), mean ± SD	2.67 ± 0.27 (*n* = 32)	2.81 ± 0.33 (*n* = 50)	0.065
PTH (pg/ml), mean ± SD	148.3 ± 118.5 (*n* = 32)	183.6 ± 182.4 (*n* = 50)	0.334
Histopathological findings			
Size (mm), mean ± SD	13.3 ± 6.7 (*n* = 24)	17.6 ± 7.4 (*n* = 47)	0.021
Weight (g), mean ± SD	1.19 ± 1.35 (*n* = 18)	2.09 ± 1.89 (*n* = 34)	0.080

**Table 3:** Histopathological findings (size and weight) in parathyroid adenomas from PET/CT-positive and SPECT/CT-positive patients and PET/CT-positive and SPECT/CT-negative patients

Histopathological findings	PET/CT-positive and SPECT/CT-positive	PET/CT-positive and SPECT/CT-negative	*p* value
Size (mm), mean ± SD	17.6 ± 7.4 (*n* = 47)	13.0 ± 6.6 (*n* = 23)	0.013
Weight (g), mean ± SD	2.09 ± 1.89 (*n* = 34)	1.24 ± 1.38 (*n* = 17)	0.106

A total of 79 parathyroid adenomas were detected, one in 59 patients, two in 8 patients and four in 1 patient. The locations of the detected adenomas are presented in Table 4. In addition, histopathological findings revealed one adenoma and three hyperplastic glands in one patient and one single parathyroid hyperplastic gland in one other patient. Overall, six false-positive lesions were seen in six patients in this study: four hyperplastic glands (in two patients), one hyperplastic gland in one patient with a normal histopathology result, and three hyperplastic glands in three patients who showed normalization of laboratory findings on the follow-up examinations without surgery.

**Table 4:** Locations of histologically verified adenomas on ^18^F-fluorocholine PET/CT

Location	Number
Right upper thyroid	11
Right lower thyroid	33
Left upper thyroid	9
Left lower thyroid	22
Intrathyroidal	2
Ectopic	2

The sensitivity, specificity, positive predictive value, negative predictive value and overall accuracy of ^18^F-fluorocholine PET/CT in the detection of parathyroid adenoma were 93.7%, 96.0%, 90.2%, 97.4% and 95.3%, respectively. Corresponding values for ^99m^Tc-MIBI/tetrofosmin SPECT/CT were 60.8%, 98.5%, 94.1%, 86.3% and 87.7%, respectively. The performance of each modality is presented in Table 5.

**Table 5:** Lesion-based sensitivity, specificity, positive predictive value (PPV), negative predictive value (NPV) and accuracy of each imaging modality based on true-positive, true-negative, false-positive and false-negative results

imaging modality	True-positive	True-negative	False-positive	False-negative	Sensitivity (%)	Specificity (%)	PPV (%)	NPV (%)	Accuracy (%)
^18^F-Fluorocholine PET/CT	74	190	8	5	93.7	96.0	90.2	97.4	95.3
^99m^Tc-MIBI/tetrofosmin SPECT/CT	48	195	3	31	60.8	98.5	94.1	86.3	87.7

Although ^18^F-fluorocholine PET-positive adenomatous lesions showed higher SUVmax values in the semiquantitative analysis than hyperplastic glands (6.80 ± 3.78 vs. 4.53 ± 0.40), the difference was not significant (*p* = 0.236). Nevertheless, in the logistic regression analysis SUVmax was the most significant factor predicting the final histopathology in contrast to preoperative serum PTH level (*p* = 0.040 vs. *p* = 0.159, respectively). Furthermore, Fisher’s exact test showed a significant association between positive ^18^F-fluorocholine PET/CT findings and the histopathological result for parathyroid adenoma (*p* = 0.003). However, there was no significant association between ^99m^Tc-MIBI/tetrofosmin SPECT/CT positivity and the histopathological findings (*p* = 0.247).

## Discussion

PHPT is newly diagnosed in 100,000 patients in the USA per year and occurs in 0.2–0.5% of the population [14]. There is currently increasing interest in MIP as the optimal surgical approach to the treatment of PHPT. This technique offers several advantages including the ability to use local or general anaesthesia and a reduced risk of recurrent or persistent hyperparathyroidism, a lower incidence and severity of symptomatic and biochemical hypocalcaemia, shorter operative time and similar results to bilateral exploration with better cosmetic outcome, and a favourable operative field in patients who require reoperation for recurrent disease [15–20]. However, accurate diagnosis and correct localization are mandatory to obtain optimal treatment results with this surgical method [5].

^99m^Tc-MIBI/tetrofosmin SPECT/CT is the current modality of choice for detection and localization of parathyroid adenoma [14, 21]. However, some studies have shown a sensitivity of about 71%, leading to false-negative results in one-third of patients with biochemical evidence of PHPT. Moreover, this approach has limited value in multiglandular disease, which is the cause of hyperparathyroidism in 5–15% of cases and is therefore associated with the risk of surgical failure [22].

**Fig. 1:**
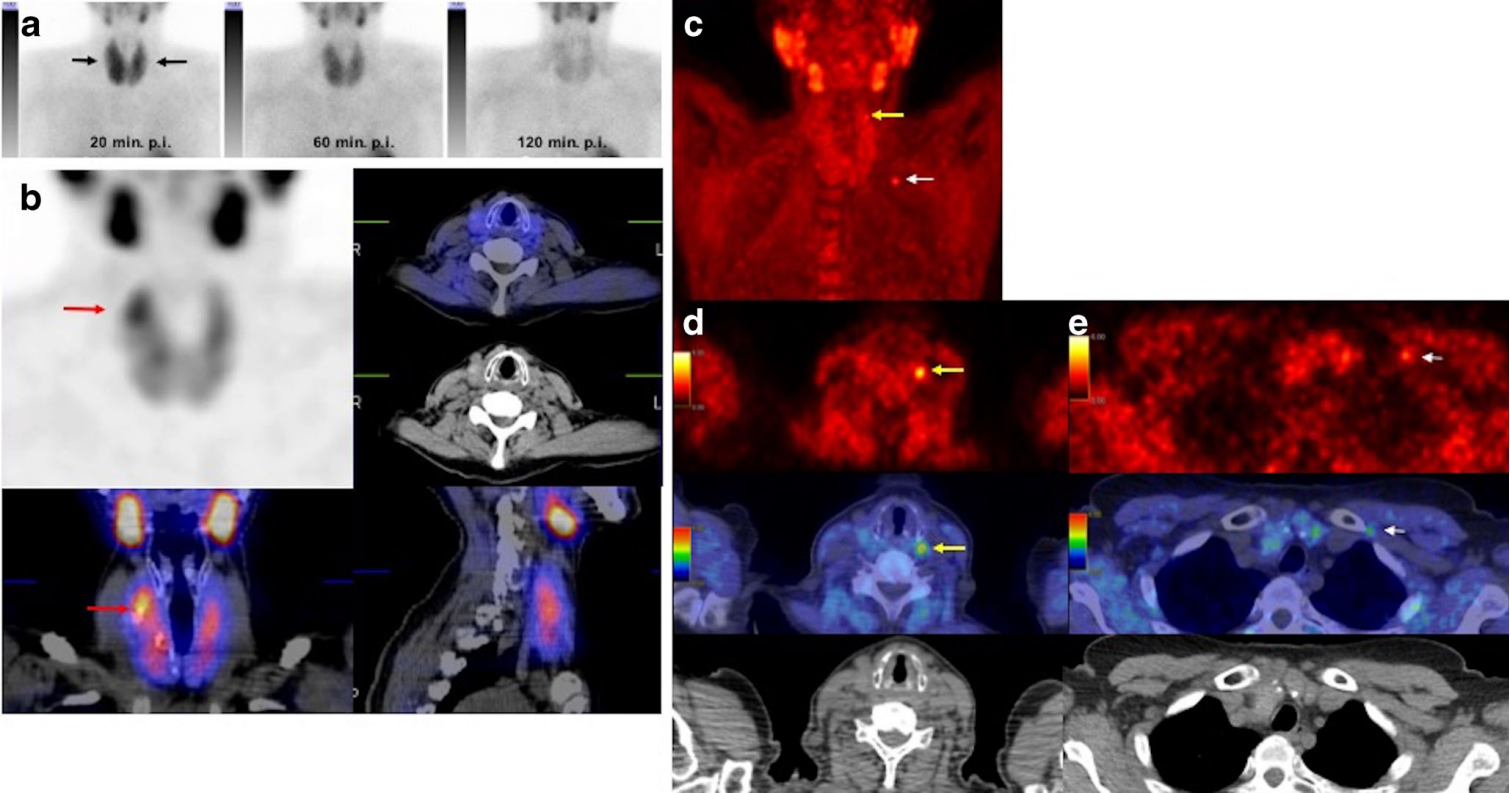
^99m^Tc-MIBI planar scintigraphy images (**a**), ^99m^Tc-MIBI SPECT/CT images (**b**) and ^18^F-fluorocholine PET/CT images (**c–e**) in a 48-year-old patient with elevated serum PTH and calcium levels of 91.29 pg/ml and 2.87 mmol/l, respectively. **a** Anterior ^99m^Tc-MIBI planar images show diffuse nonhomogeneous tracer uptake in the enlarged thyroid gland in the image 20 min after injection (20 min p.i. *arrows*) with regular wash-in seen on the 60-min image (60 min p.i.) and 120-min image (120 min p.i.) without any evidence of focal tracer retention suggestive of parathyroid adenoma. **b** ^99m^Tc-MIBI SPECT/CT images (60 min after injection) also show no evidence of pathological uptake suggestive of parathyroid adenoma. The focal tracer uptake in the upper part of the right thyroid is suggestive of thyroid adenoma (*arrows*). **c–e** ^18^F-Fluorocholine PET/CT images: maximum intensity projection (MIP) image (**c**) and transaxial PET images (**d** *top*), PET/CT fusion images (**d** *middle*) and CT images (**d** *bottom*) show marked focal tracer uptake in the upper part of the left thyroid suggestive of parathyroid adenoma (*yellow arrows*) which was confirmed on histopathology (size 9 mm, weight <1 g). Focal tracer uptake is seen in the left clavicular region on the MIP image (**c** *white arrow*) which is confirmed as nonspecific vascular uptake on the delayed images (**e** *arrows*)

**Fig. 2:**
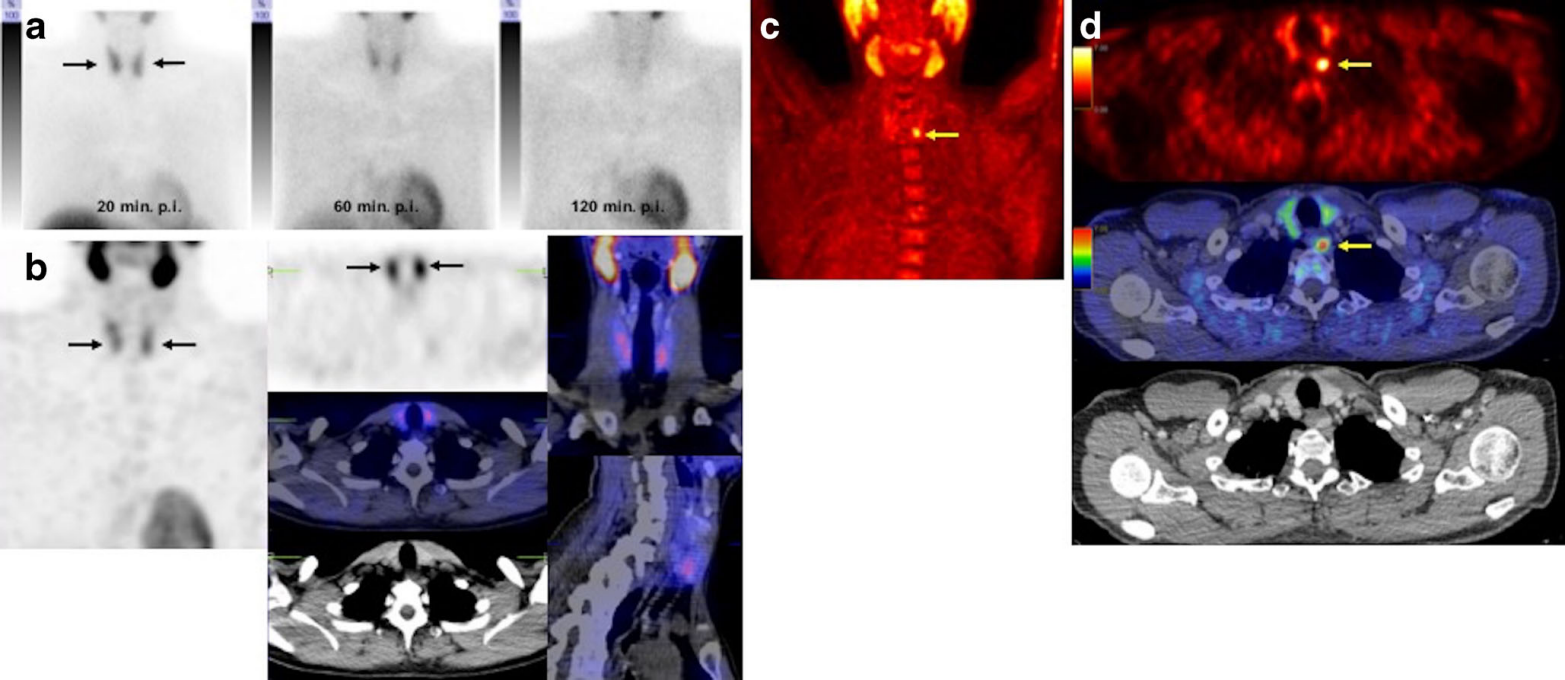
^99m^Tc-MIBI planar scintigraphy images (**a**), ^99m^Tc-MIBI SPECT/CT images (**b**) and ^18^F-fluorocholine PET/CT images (**c**, **d**) in a 67-year-old woman with an elevated serum PTH level of 108.4 pg/ml and a borderline serum calcium level of 2.51 mmol/l. **a** Anterior ^99m^Tc-MIBI planar images show homogeneous tracer uptake in the thyroid gland in the image 20 min after injection (20 min p.i. *arrows*) with regular wash-in seen on the 60-min image (60 min p.i.) and 120-min image (120 min p.i.) without any evidence of focal tracer retention suggestive of parathyroid adenoma. **b** ^99m^Tc-MIBI SPECT/CT images (60 min after injection) also show homogeneous tracer uptake in the thyroid gland (*arrows*), without any evidence of pathological uptake suggestive of parathyroid adenoma. **c**, **d** ^18^F-Fluorocholine PET/CT images: maximum intensity projection (MIP) image (**c**) and transaxial PET image (**d** *top*), PET/CT fusion image (**d** *middle*) and CT image (**d** *bottom*) show marked focal tracer uptake in the lower part of the left thyroid suggestive of parathyroid adenoma (*arrows*)

Recently, ^18^F-fluorocholine PET/CT has been introduced as a potential modality for the assessment of patients with PHPT [23–25]. Upregulation of phospholipid/Ca^2+^-dependent choline kinase has been shown to be related to PTH secretion in parathyroid adenoma [26]. In this study, ^18^F-fluorocholine PET/CT showed significantly higher sensitivity than ^99m^Tc-MIBI/tetrofosmin SPECT/CT (93.7% vs. 60.8%; Figs. 1 and 2). Our results are in accordance with those of previously published pilot studies [23, 24]. Lezaic et al. [23] compared the two modalities and found an overall lesion-based sensitivity of 92% for ^18^F-fluorocholine PET/CT which was markedly greater than that for ^99m^Tc-MIBI SPECT, which showed a sensitivity of 64%. In another pilot study by Michaud et al. [24], ^18^F-fluorocholine PET/CT proved to be more sensitive than dual phase/dual isotope scintigraphy in a lesion-based analysis. The remarkably low sensitivity of ^99m^Tc-MIBI/tetrofosmin SPECT/CT in comparison with that of ^18^F-fluorocholine PET/CT in the detection of parathyroid adenoma may be related to early diagnosis of PHPT and the small size of the adenomas.

A recent meta-analysis showed an estimated pooled sensitivity of 86% (per-patient analysis) for dual-phase ^99m^Tc-MIBI SPECT/CT in the detection of parathyroid adenoma [13]. However, published studies have mainly evaluated ^99m^Tc-MIBI SPECT/CT alone without comparing it with an advanced functional modality. Furthermore, clinical and laboratory parameters, the phase of disease at the time of imaging, whether the SPECT/CT imaging was dual-phase or single-phase, and the patient population are factors that may affect the sensitivity of this modality in the assessment of PHPT.

In addition, ^18^F-fluorocholine PET/CT was able to detect parathyroid adenoma in 23 patients in whom ^99m^Tc-MIBI SPECT/CT was negative; thus, it could be a better guide to choosing the less-invasive MIP as the surgical technique rather than bilateral neck exploration. Furthermore, ^18^F-fluorocholine PET/CT offers technical advantages over ^99m^Tc-MIBI SPECT/CT, mainly in relation to its better spatial resolution and shorter total imaging time (20 min vs. 120 min) as well as exposure of patients to less radiation especially with modern scanners [24, 25]. Although, ^18^F-fluorocholine PET/CT showed better performance than ^99m^Tc-MIBI scintigraphy in this study, whether it can be recommended as first-line imaging needs to be evaluated in future studies particularly in comparison with dual-isotope subtraction and dual-time ^99m^Tc-MIBI SPECT/CT methods. However, in six patients, ^18^F-fluorocholine PET/CT was negative. In three of these patients, serum PTH and calcium levels had normalized on the follow-up studies (true-negative), and in the other three patients (4%) ^18^F-fluorocholine PET/CT failed to detect parathyroid lesions (false-negative). This may have been related to the small size or pathological characteristics of the adenomas including a low number of adenoma-like cells corresponding to a low functional status or a fairly low number of oxyphilic cells.

^11^C-Methionine is the PET tracer that has been most investigated for the assessment of PHPT in the last decade [22, 27–29]. The sensitivity and specificity found in this study were better than the reported pooled sensitivity and specificity of 86% and 86%, respectively, found in a per patient-based analysis of ^11^C-methionine PET/CT in the evaluation of patients with PHPT and negative ^99m^Tc-MIBI SPECT [30]. Moreover, the short half-life of ^11^C limits its wide commercial use.

**Fig. 3:**
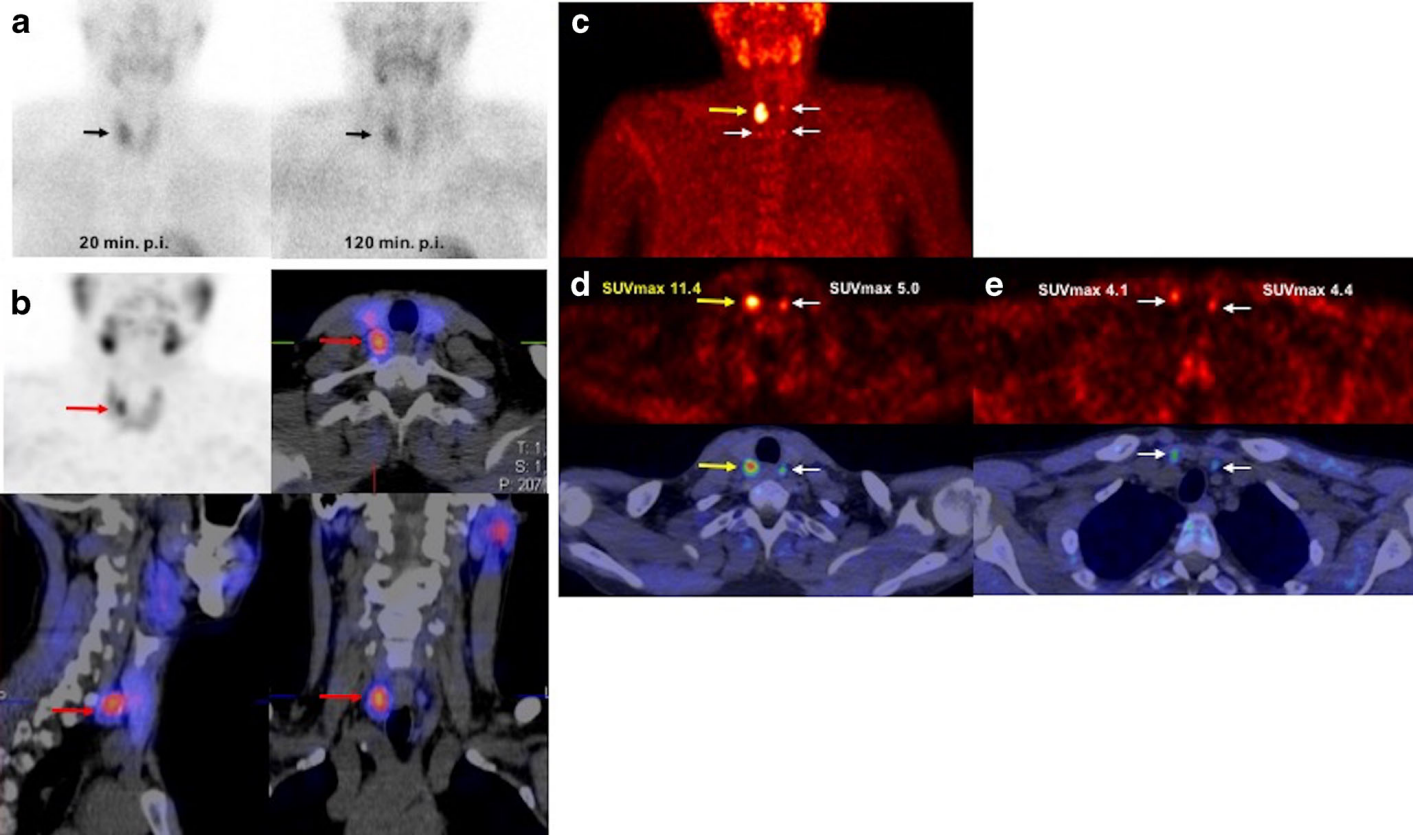
^99m^Tc-MIBI planar scintigraphy images (**a**), ^99m^Tc-MIBI SPECT/CT images (**b**) and ^18^F-fluorocholine PET/CT images (**c–e**) in a 35-year-old man with elevated serum PTH and calcium levels of 512.0 pg/ml and 2.82 mmol/l, respectively. **a** Anterior ^99m^Tc-MIBI planar images show nonhomogeneous tracer uptake in the thyroid glands with focal uptake on the right side in the image 20 min after injection (20 min p.i. *arrows*) with irregular wash-in in the 120-min image (120 min p.i.) and evidence of focal tracer retention suggestive of thyroid or parathyroid adenoma. **b** ^99m^Tc-tetrofosmin SPECT/CT images (120 min after injection) show pathological focal uptake in the posterior part of the right thyroid lobe suggestive of parathyroid adenoma. **c–e** ^18^F-Fluorocholine PET/CT images: maximum intensity projection (MIP) image (**c**) shows focal tracer uptake in the upper posterior part of the right thyroid lobe (*yellow arrows*) with three small areas of mild tracer uptake in the other parathyroid glands (*white arrows*) which was found to be hyperplastic parathyroid glands on histopathology; **d** transaxial PET image (*top*) and PET/CT fusion image (*bottom*) show marked focal tracer uptake in the upper part of the right thyroid suggestive of parathyroid adenoma (*yellow arrows*) and a small mild focal lesion in the upper part of the left thyroid suggestive of parathyroid hyperplasia (*white arrows*); **e** transaxial PET image (*top*) and PET/CT fusion image (*bottom*) show marked small mild focal lesions on the lower part of both thyroid lobes suggestive of parathyroid hyperplasia

In this study, we also assessed the impact of semiquantitative analysis on the differentiation between parathyroid adenoma and hyperplasia. Adenomatous lesions showed a higher SUVmax than hyperplastic glands (mean 6.80 ± 3.78 vs. 4.53 ± 0.40; Fig. 3), but the difference was not statistically significant (*p* = 0.236). This may have been because the analysis was affected by the small number of lesions (four lesions) labelled as hyperplasia on histopathology. Nevertheless, in a previous study by Michaud et al. with a higher number of hyperplastic parathyroid glands than adenomas (15 and 9, respectively), the authors also found this trend for greater uptake in adenomas (mean SUVmax 4.6) than in hyperplasia (mean SUVmax 3.5), but they were also unable to find a meaningful cut-off value for differentiating the two categories [24].

In this research, the prevalences of solitary parathyroid adenoma, double adenomas and four-gland adenomas were 74% (59/79), 20% (16/79) and 5% (4/79), respectively. In contrast to the findings of previous studies, the prevalence of parathyroid hyperplasia was lower in our study (overall four hyperplastic glands in two patients) [2, 24]. This may be related to the different histopathological definitions and criteria for the differentiation of parathyroid adenoma from hyperplasia in various departments. Adenomas and hyperplasia both mainly consist of chief cells, and are devoid of adipocytes and cytoplasmic lipid. The involvement of a single gland is the widely accepted criterion for the diagnosis of adenoma based on macroscopic and/or microscopic findings in the remaining associated glands [31, 32]. The accurate detection and localization of parathyroid adenomas and performing MIP, that led to a decrease in iPTH levels of more than 50% in 78% of our patients, may be the reason for the low number of parathyroid hyperplastic glands.

Finally, the multivariate analysis showed that the size of the adenomas was significantly different between patients negative on ^99m^Tc-MIBI/tetrofosmin SPECT/CT and those positive on ^18^F-fluorocholine PET/CT. However, there was no meaningful difference in other laboratory and histopathological findings including serum calcium and PTH or the weight of the adenomas between these two groups.

### Limitations of study

In 40% of patients we used ^99m^Tc-tetrofosmin for SPECT/CT examinations which may have affected the sensitivity of this modality [9]. However, in view of the equal detection efficiencies of ^99m^Tc-MIBI and ^99m^Tc-tetrofosmin reported in the literature, this may have caused no meaningful bias in the results of this study [8, 9, 33]. Early SPECT/CT was not performed in this study for ethical reasons and exposure of patients to additional radiation. This may have reduced the sensitivity of ^99m^Tc-MIBI/tetrofosmin SPECT/CT in the detection of small hyperactive parathyroid tissues with rapid washout and may have been a methodological limitation of this study that could have caused a bias in the reported sensitivity. Due to the homogeneity of the patient population of this study, we consider that direct comparison of the imaging modalities used in each study centre would have had no significant influence on the results. Therefore, the data were not analysed separately.

### Conclusion

^18^F-Fluorocholine PET/CT was shown to be clearly superior to ^99m^Tc-MIBI/tetrofosmin SPECT/CT in this prospective study in a large patient population. It shows very promising potential for first-line functional imaging for the early detection and localization of small parathyroid adenomas in patients with PHPT. Semiquantitative analysis may provide additional information in differentiating parathyroid adenoma from hyperplasia, but further evaluation is warranted.
